# Multidisciplinary wound care in an elderly patient with extensive bullous pemphigoid: a case report

**DOI:** 10.3389/fmed.2026.1883790

**Published:** 2026-09-03

**Authors:** Xiaoyan Chen, Masoud Mohammadnezhad, Yuhua Zhou, Yubing Qin

**Affiliations:** 1 Department of Neurology, Shantou Central Hospital, Shantou, China; 2 School of Nursing and Midwifery, Birmingham City University, Birmingham, United Kingdom; 3 Department of Public Health, Daffodil International University (DIU), Daffodil Smart City (DSC), Birulia, Savar, Dhaka, Bangladesh; 4 Department of Nursing, Shantou Central Hospital, Shantou, China

**Keywords:** bullous pemphigoid, individualised cut-to-fit dressing, moist wound healing, multidisciplinary collaboration, older adult, wound nursing care

## Abstract

This case report evaluated the effectiveness of individualised cut-to-fit dressings, guided by moist wound healing principles, in managing multiple refractory wounds in an elderly patient with bullous pemphigoid. A multidisciplinary team collaborated with a wound specialist nurse who tailored and combined dressings according to wound exudate levels, pressure exposure, and skin tension, while reinforcing comprehensive assessment, medication management, skin protection, and health education. During hospitalisation, personalised moist dressings protected multiple wound sites, reduced dressing waste and financial burden, decreased exudate and pain, and promoted gradual epithelialisation without infection or major complications. Post-discharge follow-up showed stable systemic and local recovery with sustained wound healing. These findings suggest that multidisciplinary collaboration combined with individualised cut-to-fit dressings and meticulous wound management can effectively enhance healing of complex bullous pemphigoid wounds in older adults, reduce infection risk, and achieve a balance between therapeutic benefit and cost control.

## 1 Introduction

Bullous pemphigoid (BP) is an autoimmune subepidermal blistering disease with a recognised genetic predisposition, predominantly affecting older adults with age-related immune decline (1). It typically presents with generalised pruritus and tense blisters (2). The overall incidence of BP has been reported as 0.1138 per 10,000 person-years (3). The incidence of BP demonstrated notable geographical variation, with rates of 0.2397 per 10,000 person-years in North America, 0.1444 per 10,000 person-years in Europe, 0.0800 per 10,000 person-years in Asia, and 0.0342 per 10,000 person-years in Africa (3). In addition to regional differences, incidence rose substantially with age, from 0.0313 per 10,000 person-years in individuals aged 60–69 years to 0.1154 in those aged 70–79 years and 0.1913 in those aged ≥80 years (3). This trend aligns with epidemiological data from the United Kingdom, where the median age at BP onset reaches 80 years (4). BP not only compromises skin integrity and quality of life, causing pain, pruritus and functional limitation, but also increases the risk of hospitalisation and mortality due to secondary infection, electrolyte disturbances and complications associated with prolonged immobility (5). In older adults, impaired skin barrier function, restricted mobility, pain and pruritus all contribute to delayed wound healing (6). Among elderly patients with BP, the disease itself often progresses rapidly and involves extensive skin damage (7). In addition, reduced physiological reserves, a high burden of comorbidities and frailty further complicate both treatment and nursing care (8).

This case report aims to explore the feasibility and effectiveness of an individualised wound care strategy in an elderly female patient with bullous pemphigoid presenting with multiple sites of skin breakdown. We describe a tailored dressing approach using cut-to-fit materials suitable for multi-site lesions, developed to address limitations of conventional dressings in terms of coverage, conformity, and fixation. This approach may offer a practical, reproducible management option in clinical settings.

## 2 Case presentation

A 91-year-old female patient, Ms. Chen, was admitted to our department on 30 August 2025 with a three-month history of recurrent widespread bullae, 7 days of skin ulceration and 4 days of left-sided limb weakness. The admission diagnoses included cerebral infarction, hypertension, widespread cutaneous bullae, status post left lower-leg fracture fixation, and hypokalaemia. On physical examination at admission, the patient’s temperature was 36.7 °C, heart rate 103 beats/min, respiratory rate 21 breaths/min, and blood pressure 132/93 mmHg.

**Figure 1:**
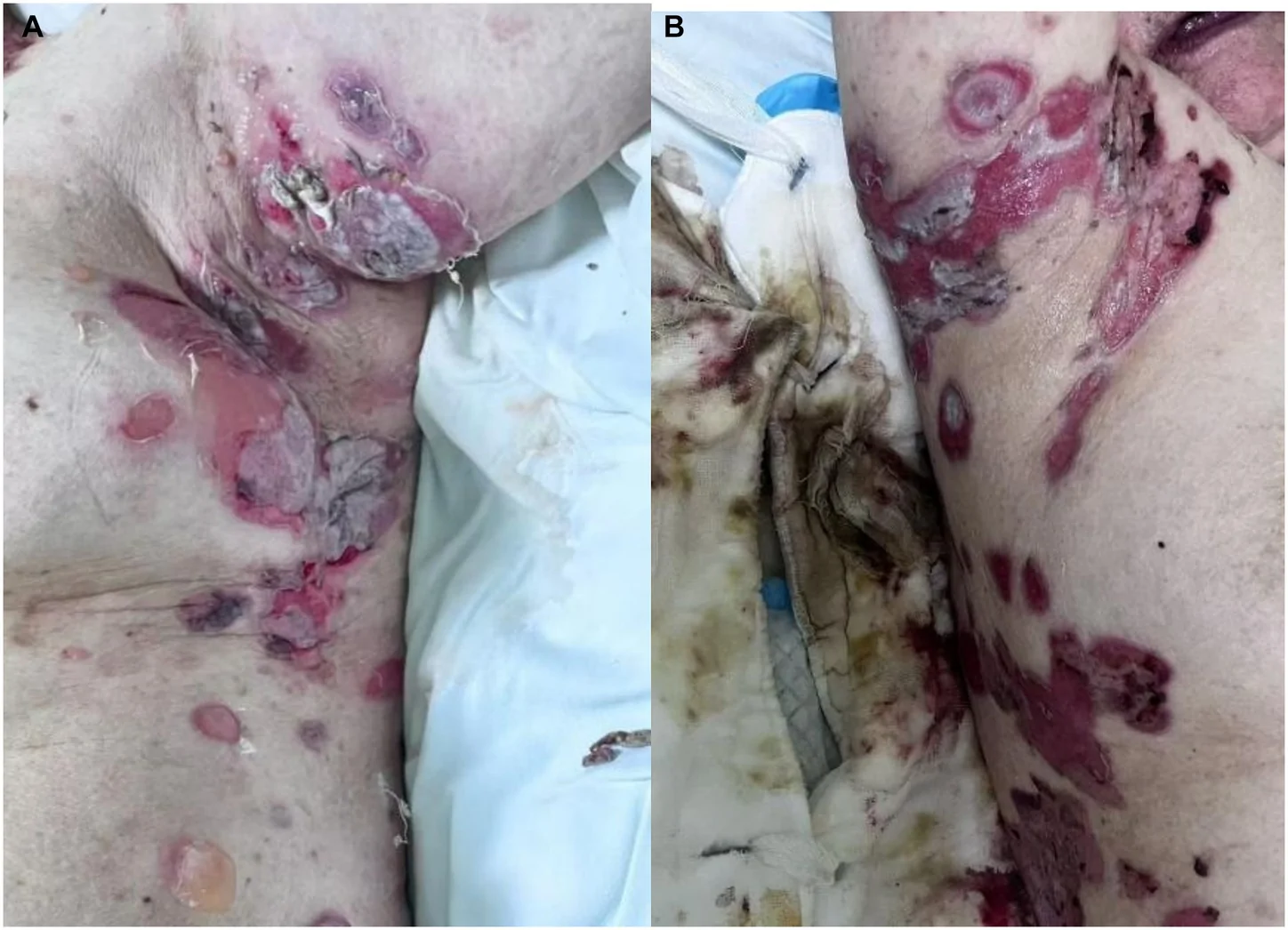
**(A,B)** Skin lesions on admission.

The patient was alert and oriented, but in generally poor condition with evidence of malnutrition. Severe pain was reported due to ulceration secondary to blister rupture. Approximately 40% of the total body surface area was affected by cutaneous ulceration, involving multiple anatomical sites including the bilateral axillae, forearms, inguinal regions, lower limbs, back, neck, chest and scapular areas (Figures 1A,B). The extensive ulcerated areas exhibited haemorrhagic exudate, with some regions covered by dark red crusts. The Bullous Pemphigoid Disease Area Index (BPDAI) score was 180. Laboratory investigations revealed a high-sensitivity C-reactive protein level of 19.66 mg/L, serum potassium of 3.42 mmol/L, and glycated haemoglobin (HbA1c) of 6.1%. Urinalysis showed 1 + proteinuria. Wound culture yielded a small number of bacilli. Following admission, a dermatology consultation was requested. Skin biopsy for routine histopathological examination demonstrated a subepidermal blister with abundant eosinophilic infiltration within the blister cavity and moderate perivascular infiltration of eosinophils, together with scattered lymphocytes and neutrophils in the superficial dermis. Direct immunofluorescence revealed linear C3 deposition along the basement membrane zone, whereas IgA, IgG, and IgM were negative. Serological testing showed a BP180 antibody level >400.00 U/mL, while BP230 antibody testing was negative, confirming the diagnosis of bullous pemphigoid. Continuous electrocardiographic monitoring was initiated to observe vital signs and blood glucose levels, and supplemental oxygen was administered to improve cerebral perfusion. A urinary catheter was placed to monitor 24-h urine output. The patient received immunomodulatory therapy with dupilumab, and ceftriaxone was administered for infection control (9). Analgesia was provided with oral lofexidine and pregabalin, and potassium supplementation was given as sustained-release potassium chloride tablets. Corticosteroid therapy with prednisone acetate was initiated, and intravenous immunoglobulin was administered to enhance immune function (10). Additional supportive therapies were also implemented, including neurotrophic agents, calcium supplementation, gastro- and renoprotective measures, and nutritional support.

## 3 Nursing interventions

### 3.1 Skin care and wound management

The patient presented with numerous haemorrhagic bullae of varying sizes distributed across the body, involving the axillae, forearms, bilateral groins, lower limbs, back and scapular regions. Marked tenderness was elicited on palpation, and even minimal contact provoked severe pain, making it difficult for the patient to lie flat at night. A moist wound-healing strategy was adhered to throughout the care process (11). Guided by six core principles of pain-free intervention, atraumatic care, epidermal preservation, continuous drainage, high absorbency and friction minimisation; an individualised wound management regimen was implemented (12).

#### 3.1.1 Atraumatic and gentle debridement

Irrigation was performed using a low-pressure non-alcoholic povidone-iodine (Type III; an aqueous povidone-iodine formulation commonly used in China) to remove surface crusts and exudate, achieve antisepsis, and maintain wound moisture (13). Following irrigation, the wound surface was gently wiped with sterile water for injection to minimise residual iodine-related irritation.

#### 3.1.2 Targeted pressure offloading

Small blisters with a diameter of <1 cm were left intact to minimise frictional trauma and allow for spontaneous resolution. Blisters with diameters of ≥1 cm and ≤2 cm were disinfected with a non-alcoholic povidone-iodine (type III). Using a 1 mL sterile syringe needle, the needle was inserted parallel to the base of the blister and the skin surface, and the fluid was gently aspirated (14). Care was taken to ensure that the needle tip always remained within the blister cavity, avoiding repeated punctures. After aspiration, gentle pressure was applied to the blister roof using the fingertips, allowing it to adhere evenly to the wound bed and form a natural “biological dressing.” For blisters with a diameter of ≥2 cm, a 2–3 mm triangular incision was made at the lowest point using sterile small scissors. Gentle pressure was applied with sterile cotton swabs during the procedure to facilitate drainage, ensuring multiple channels through a single opening to prevent adhesion of the wound edges. If the blister roof was folded, sterile forceps were used to gently flatten it, thereby restoring its barrier function.

#### 3.1.3 High absorbency wound dressing with secure fixation

Immediately after aspiration, a thin layer of hydrocolloid gel containing hyaluronic acid was applied to the wound bed to maintain moisture. The wound was then covered with a layer of petrolatum gauze to prevent adherence. Sterile oversized cotton pads were cut and shaped according to body contours: “butterfly”-shaped pads for the axillae, “V”-shaped pads for the inguinal regions, and tubular pads for the upper and lower limbs (15, 16). Holes were made at the edges of each pad, and breathable straps were used to interconnect them both longitudinally and transversely, forming a short-sleeved vest-like garment with a front opening for daily inspection and dressing changes. The complete vest was soft, breathable, capable of absorbing substantial exudate, and maintained a dry surface for up to 24 h. The absorbent cotton vest was routinely replaced every 24–48 h, depending on the amount of exudate, and was changed earlier if it became saturated or displaced. Routine daily dressing changes were therefore unnecessary, as the dressing maintained an optimal wound environment while reducing pain and trauma associated with frequent dressing replacement. An elastic abdominal band was applied to provide secondary fixation, preventing displacement of the dressing during repositioning or movement.

#### 3.1.4 Continuous assessment and documentation

During each shift, the characteristics of blister fluid, drainage volume, colour of the blister roof, and temperature of the surrounding skin were recorded (17). If closure of the incision or accumulation of exudate was observed, a 1 mm extension was made adjacent to the original incision to ensure continuous, low-resistance drainage.

Through the meticulous, stepwise, and modular nursing approach, the epidermal barrier was maximally preserved while achieving effective pressure offloading, sustained moisture, and high-absorbency fixation.

### 3.2 Infection prevention

Visitor access was restricted, and the patient’s room was disinfected with ultraviolet light once daily for 30 min to 1 h. A designated caregiver was assigned to minimise cross-infection. The patient’s nails were trimmed to prevent accidental rupture of blister roofs and subsequent infection. Bed linens were kept clean and flat, changed daily, and all medical textiles were double-bagged, clearly labelled, and washed and disinfected separately; clothing and other personal items were also cleaned using dedicated containers. Strict adherence to aseptic technique and hand hygiene was enforced among healthcare personnel, with standard precautions and isolation measures implemented. Blister fluid was aspirated using sterile syringes, with a gentle technique to maintain the integrity of the blister roofs and avoid friction or rupture. Vital signs and inflammatory markers were monitored regularly, and the patient was assessed for new skin lesions, fever, or erythema and swelling at lower limb lesion sites (18).

### 3.3 Nutritional support

Considering the patient’s advanced age, extensive skin lesions, increased wound exudate, and elevated basal energy expenditure, a diet of soft, easily digestible, and bland foods was provided (19). Intake of foods high in salt, fat, and cholesterol, as well as spicy or hard-to-digest items, was restricted to reduce gastrointestinal burden and prevent exacerbation of underlying conditions. To meet the nutritional requirements for tissue repair, the patient was encouraged to consume high-protein and high-fibre foods, including fish, lean meat, eggs, dairy products, and soy-based products, to promote collagen synthesis and wound healing. The patient and family were guided on appropriate meal composition, with attention to energy and protein balance, and advised to take small, frequent meals to improve intake tolerance.

### 3.4 Psychosocial support

Owing to extensive skin lesions and the prolonged course of the illness, the patient perceives her prospects for recovery as limited. The death of her husband from pemphigus has contributed to heightened tension, anxiety, and fear, not only in the patient but also within her family. Interactions between the patient and family members are often coloured by negative emotions, further exacerbating her psychological burden.

Supportive psychological care was therefore incorporated throughout the patient’s hospitalisation. The nursing team conducted regular psychological assessments through daily communication, encouraging the patient to express her fears, concerns, and emotional distress. Individualised emotional support and reassurance were provided, while realistic short-term treatment goals were established to enhance her confidence in recovery and reduce feelings of helplessness.

Nurses foster patients’ confidence in treatment and self-efficacy by providing education on disease knowledge and offering regular feedback on progress. During each dressing change, they attentively listen to the patient’s reports of pain, pruritus, and other concerns, responding with empathy.

Given the emotional impact of the disease on the family, supportive psychosocial care was also extended to family members. Nurses provided education regarding the disease course, prognosis, and treatment expectations, addressed their concerns through regular communication, and encouraged positive coping strategies. Family members were advised to avoid expressing excessive anxiety or pessimism in front of the patient and were encouraged to provide consistent emotional support rather than focusing solely on the illness. Formal family therapy was not undertaken; instead, structured supportive counselling and ongoing family education were integrated into routine nursing care.

Concurrently, family members are engaged and guided to minimise negative expressions and to help establish a supportive family environment. They are also involved in simple aspects of care, enhancing their sense of participation and support.

## 4 Outcome and follow-up

**Figure 2:**
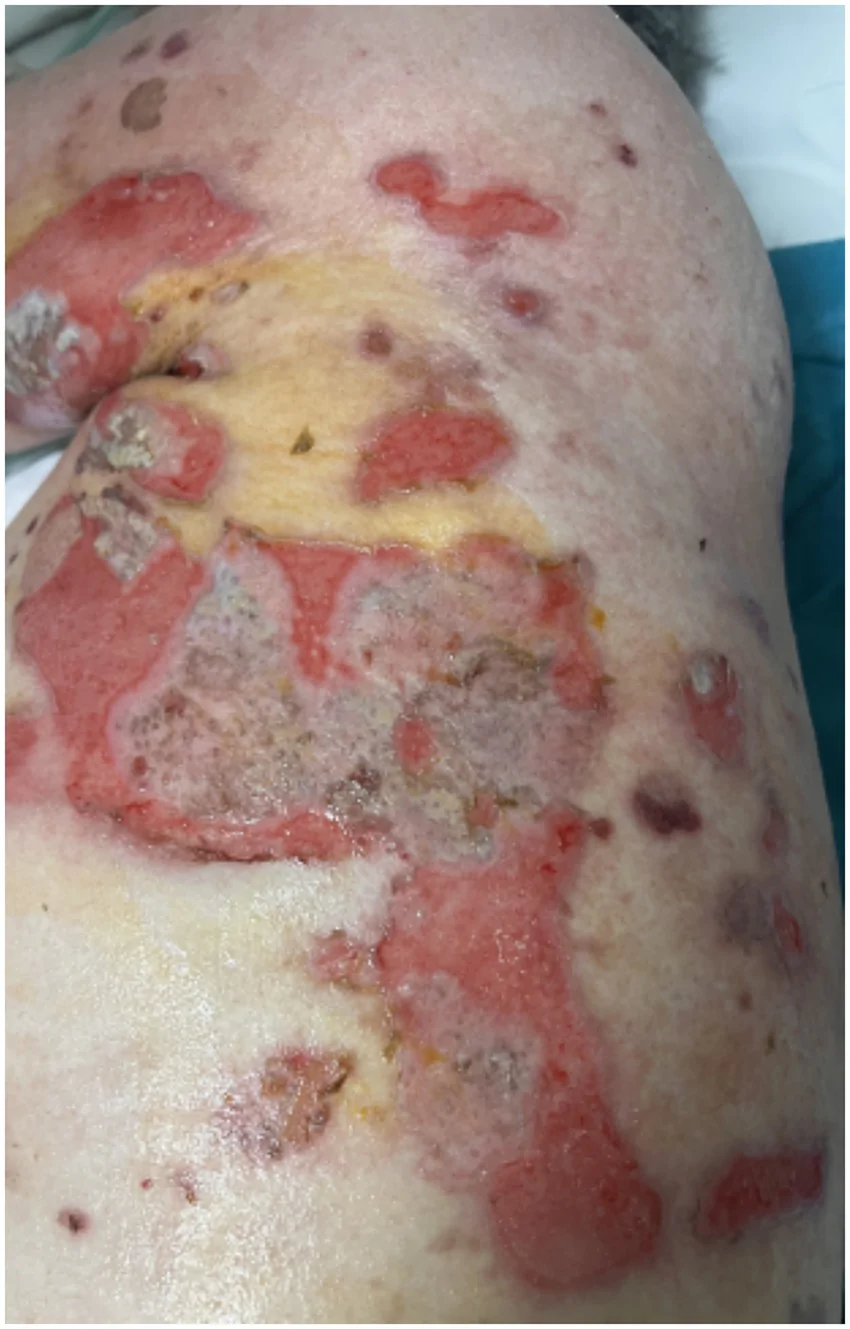
Wound healing after 10 days of hospitalisation.

**Figure 3:**
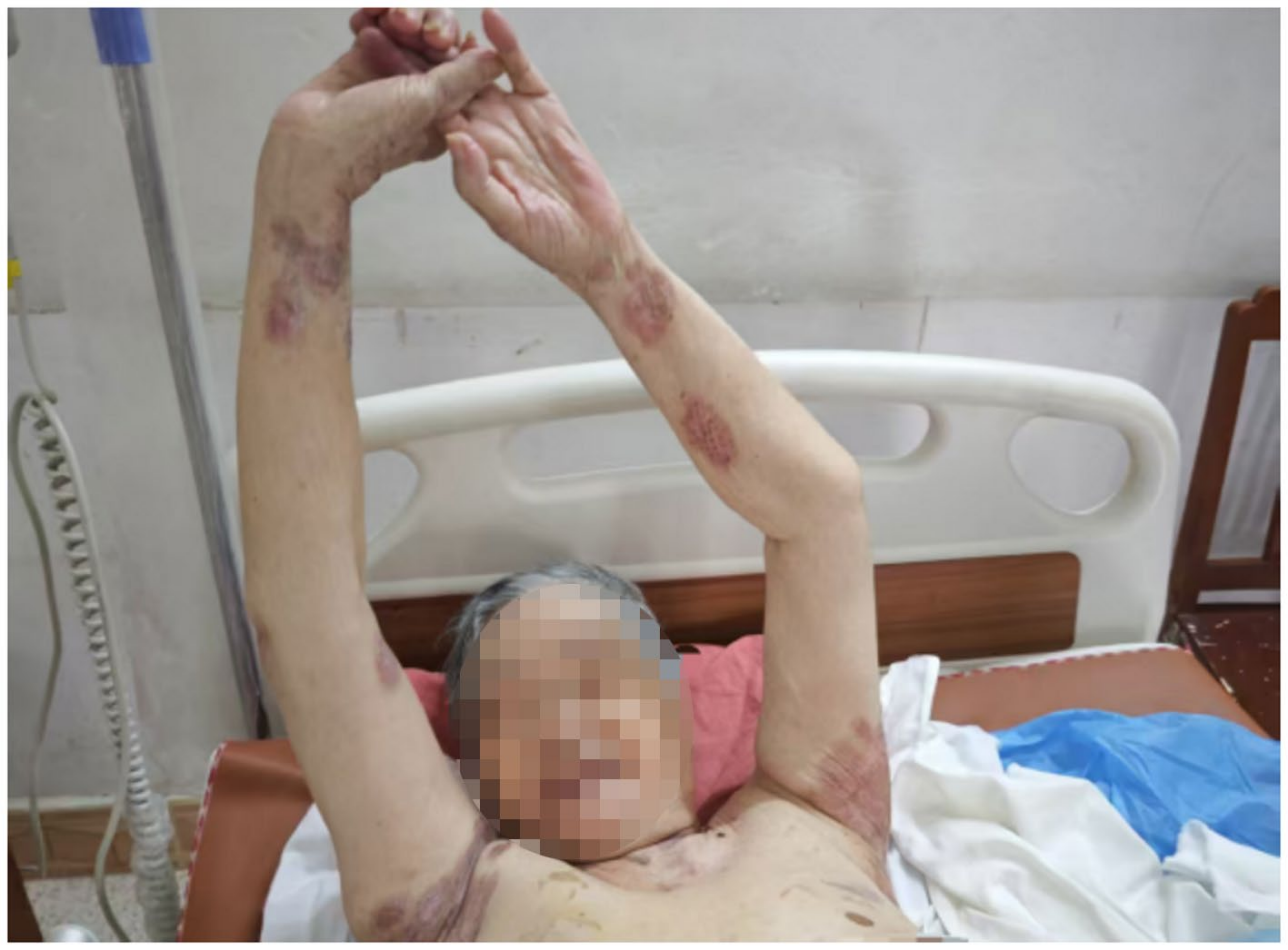
Wound healing at 20-day follow-up.

Through multidisciplinary team consultations, medical staff closely monitored the patient, promptly identifying gaps in nursing care, strengthening management, and involving wound specialist nurses in dressing changes. The patient was hospitalised for ten days in a stable condition, during which a total of six dressing changes were performed, and follow-up wound cultures were negative. Guided by the principles of moist wound healing, the nursing team implemented personalised cut-to-fit dressings and meticulous wound management, tailored to the characteristics of the patient’s multiple skin lesions. On the day of discharge, epithelialisation was observed in the wounds on the patient’s upper and lower limbs as well as the inguinal regions. Wounds beneath the axillae and on the back exhibited a healthy red granulation base, while scabs on the chest and neck were dry. No new blisters or signs of secondary infection were noted (Figure 2). Skin ulcers covered approximately 20% of the total body surface area. The patient’s pain was substantially reduced, with a pain score of 2, and the activities of daily living (ADL) score was 50, indicating partial self-care ability. After discharge, a wound care specialist nurse visited the patient at home to perform wound cleaning and dressing. Follow-up on day 20 post-discharge revealed that the original epidermal blisters had fully dried and formed scabs, the wounds had completely healed, and no new blisters or signs of secondary infection were observed (Figure 3). The patient experienced no additional complications, and the family expressed high satisfaction with the medical and nursing care provided.

## 5 Discussion

Bullous pemphigoid (BP) is a chronic, relapsing autoimmune subepidermal blistering disease that predominantly affects older adults and is commonly accompanied by multiple comorbidities (2). Poor disease awareness and suboptimal adherence to long-term treatment, together with inadequate control of chronic conditions such as hypertension, contribute to recurrent disease activity and an increased risk of serious complications, including hospitalisation for cerebral infarction (5). Therefore, healthcare professionals should enhance communication with patients and their families, provide comprehensive disease education to ensure a thorough understanding, reinforce patients’ proactive engagement in treatment, and foster determination to adhere to therapy in collaboration with the medical team. The patient in this case is an elderly woman with extensive skin lesions affecting multiple sites. The prolonged course of the disease has imposed substantial physical and psychological burdens, manifesting as marked anxiety and fear. In addition, the patient’s family lacked standard knowledge and skills in dressing changes and skin care, further increasing the complexity and risks associated with wound management. Collectively, these factors posed significant challenges for wound care, psychological support, and health education in this case.

The nursing strategy employed in this case offers several advantages. Firstly, non-alcoholic povidone-iodine (type III) was selected as the wound cleanser during dressing changes; it is less irritating and gentler than saline, thereby reducing the patient’s pain and discomfort during debridement and dressing, thereby improving tolerance and compliance (13). Secondly, for large, scattered blisters and eroded wounds, a personalised cotton pad cutting method was used to provide tailored dressings that “custom-fit” different anatomical areas. The materials are readily available and inexpensive, reducing dressing usage and hospital costs, which enhances scalability (12). Thirdly, a multidisciplinary collaboration model was implemented, led by wound care specialist nurses for wound assessment and dressing changes, with support from dermatology and nutrition departments. During hospitalisation, 60% of the patient’s medical expenses were covered by the national health insurance scheme, while the remaining 40% were paid by the patient. This approach enabled continuous dynamic wound monitoring and interventions, thereby improving the professionalism and specificity of nursing management (20).

Previous studies have reported that the use of potassium permanganate wet dressings combined with infrared irradiation, followed by coverage with silver ion dressings in patients with BP, can effectively provide anti-infective effects and promote granulation tissue formation, resulting in favourable wound healing outcomes (21). This approach is particularly advantageous for lesions with heavy exudate or a high risk of infection. However, in the practical nursing environment of this case, limitations in ward equipment, patient financial constraints, and material availability made it difficult to fully replicate the comprehensive skin repair protocol described above. Given the patient’s extensive wound distribution, complex anatomical locations, fragile skin, and heightened pain sensitivity, a more feasible and cost-effective strategy was adopted, incorporating personalised cotton pad cutting, gentle wound cleansers, and a multidisciplinary collaboration model. Despite these limitations, this approach effectively controlled exudate, alleviated pain, reduced the risk of infection, and offered benefits in terms of cost and patient adherence, demonstrating clinical applicability and potential for broader implementation.

## 6 Conclusion

This case demonstrates that, in elderly patients with BP, factors such as extensive skin lesions, heightened pain sensitivity, substantial psychological burden, and limited caregiving knowledge markedly increase the complexity of wound management. In settings constrained by equipment and resources, a comprehensive approach employing gentle wound cleansers, customised dressing cuttings, and multidisciplinary collaboration can achieve favourable outcomes in controlling exudate, alleviating pain, enhancing patient adherence, and reducing costs, thereby providing a practical nursing strategy for similar patients.

For elderly patients with BP, clinical care should prioritise: (i) standardised wound management procedures and appropriate selection of dressings; (ii) comprehensive health education for patients and their families, including training in dressing change techniques; (iii) integration of psychological support and routine emotional assessment; and (iv) promotion of multidisciplinary collaboration models, with active involvement of specialised wound care nurses. Concurrently, there is a need for more systematic data collection and comparative studies to further evaluate the effectiveness and cost-efficiency of different care models, and to establish generalisable, evidence-based nursing strategies for the BP population.

## Data Availability

The original contributions presented in the study are included in the article/supplementary material, further inquiries can be directed to the corresponding author.
